# Evaluating the Impact of Artificial Intelligence (AI) on Clinical Documentation Efficiency and Accuracy Across Clinical Settings: A Scoping Review

**DOI:** 10.7759/cureus.73994

**Published:** 2024-11-19

**Authors:** Craig Lee, Shawn Britto, Khaled Diwan

**Affiliations:** 1 General Internal Medicine, University Hospitals Plymouth NHS Trust, Plymouth, GBR

**Keywords:** ai and machine learning, artificial intelligence in medicine, electronic health record (ehr), machine learning (ml), natural language programing (nlp), scoping review, speech recognition, ward round

## Abstract

Artificial intelligence (AI) technologies (natural language processing (NLP), speech recognition (SR), and machine learning (ML)) can transform clinical documentation in healthcare. This scoping review evaluates the impact of AI on the accuracy and efficiency of clinical documentation across various clinical settings (hospital wards, emergency departments, and outpatient clinics).

We found 176 articles by applying a specific search string on Ovid. To ensure a more comprehensive search process, we also performed manual searches on PubMed and BMJ, examining any relevant references we encountered. In this way, we were able to add 46 more articles, resulting in 222 articles in total. After removing duplicates, 208 articles were screened. This led to the inclusion of 36 studies. We were mostly interested in articles discussing the impact of AI technologies, such as NLP, ML, and SR, and their accuracy and efficiency in clinical documentation. To ensure that our research reflected recent work, we focused our efforts on studies published in 2019 and beyond. This criterion was pilot-tested beforehand and necessary adjustments were made. After comparing screened articles independently, we ensured inter-rater reliability (Cohen's kappa=1.0), and data extraction was completed on these 36 articles. We conducted this study according to the Preferred Reporting Items for Systematic Reviews and Meta-Analyses (PRISMA) guidelines.

This scoping review shows improvements in clinical documentation using AI technologies, with an emphasis on accuracy and efficiency. There was a reduction in clinician workload, with the streamlining of the documentation processes. Subsequently, doctors also had more time for patient care. However, these articles also raised various challenges surrounding the use of AI in clinical settings. These challenges included the management of errors, legal liability, and integration of AI with electronic health records (EHRs). There were also some ethical concerns regarding the use of AI with patient data.

AI shows massive potential for improving the day-to-day work life of doctors across various clinical settings. However, more research is needed to address the many challenges associated with its use. Studies demonstrate improved accuracy and efficiency in clinical documentation with the use of AI. With better regulatory frameworks, implementation, and research, AI can significantly reduce the burden placed on doctors by documentation.

## Introduction and background

Introduction

In our daily ward rounds, we often think about the heavy task of documenting patient information throughout the day, whether this may be progress notes, clerking, or discharge summaries. There were days when records were all handwritten; however, we have now moved towards a digital era where electronic health records (EHRs) are the norm [1-3]. The introduction of computers completely revolutionized healthcare, making it easy for staff to share patient information where needed and providing easy access to patient histories [4-6].

As we considered these advancements, we thought about the next revolutionary change. Could artificial intelligence (AI) enhance our clinical documentation processes similarly to how computers improved healthcare? The potential for AI to reduce workload and documentation burden seems promising [7-11]. This scoping review aims to explore the impact of natural language processing (NLP), machine learning (ML), and speech recognition (SR) on the accuracy and efficiency of clinical documentation across various clinical settings, including hospital wards, emergency departments, and outpatient clinics [12-20]. By having a look at the current literature, we seek to discover how AI can support healthcare staff and improve patient care [21-25].

Definitions of Key Terms

AI: AI refers to the simulation of human intelligence processes by machines, especially computer systems. These processes include learning (the acquisition of information and rules for using the information), reasoning (using rules to reach approximate or definite conclusions), and self-correction [26,27].

NLP: NLP is a branch of AI that focuses on the interaction between computers and humans through natural language. It involves the development of algorithms and models that enable computers to process and analyze large amounts of natural language data [12-15].

ML: ML is a subset of AI that involves the use of statistical techniques to give computer systems the ability to learn from data and improve from experience without being explicitly programmed. It includes various methods such as supervised learning, unsupervised learning, and reinforcement learning [10,16,17].

SR: SR is a technology that enables the recognition and translation of spoken language into text by computers. It involves the use of algorithms to process and analyze vocal input and is commonly used in applications such as voice assistants and transcription services [16,20].

Large language models (LLMs): LLMs are a type of AI model designed to understand and generate human-like text based on vast amounts of data. These models, such as GPT-3, are trained on diverse datasets and can perform various language-related tasks, including translation, summarization, and question-answering [23,25,28-30].

Rationale

Shifting from handwritten to digital records via EHRs has created some challenges in clinical documentation, by increasing the number of administrative tasks that healthcare professionals need to perform. This detracts from patient care. There is evidence that suggests that AI can help ease the burden of these administrative tasks in clinical documentation, with the use of ML, NLP, and SR. These technologies are being used with greater efficiency and accuracy as time goes by; however, the literature does highlight the challenges of using AI in this context.

This scoping review will explore the impact of AI on clinical documentation in terms of efficiency and accuracy and look at challenges that arise while using it across various healthcare settings. A scoping review approach is suitable given the exploratory nature of the questions and the diversity of study designs, allowing for the comprehensive mapping of existing literature, identification of research gaps, and examination of complex AI interventions in clinical workflows. This review can offer further insight for stakeholders and may help guide research and integration of AI into healthcare in the future.

Methods

Study Design

This scoping review aimed to investigate the impacts of AI technologies on the accuracy and efficiency of clinical documentation across different clinical settings. Our primary research question was "What are the impacts of AI technologies on the accuracy and efficiency of clinical documentation in various clinical settings?".

Search Strategy

A literature search was done on Ovid, and the search string used to identify pertinent articles is shown below in Table 1.

**Table 1:** Search strategy for identifying literature on AI in clinical documentation AI: artificial intelligence

Step	Search string
1	(“artificial intelligence” or “AI” or “natural language processing” or “machine learning”).mp.
2	exp Artificial Intelligence/
3	1 or 2
4	((clinical adj document*) or (medical adj document*) or (health adj record*) or (patient adj record*)).mp.
5	exp medical records/ or exp electronic health records/
6	4 or 5
7	3 and 6
8	7 and (“language model” or “LM” or “SR” or “speech recognition”).mp.
9	limit 8 to yr=“2019 - Current”

This search strategy yielded 176 articles. We then combined the Ovid search with manual searches in PubMed and BMJ, as well as reference examination, identifying an additional 46 articles. This process resulted in a total of 222 articles. After removing 14 duplicates, we screened 208 articles. Following this, we excluded 154 articles and assessed 54 full texts for eligibility, ultimately narrowing our selection down to 36 studies for inclusion in our scoping review.

Inclusion and Exclusion Criteria

Specific inclusion and exclusion criteria were used to select our 36 articles. The inclusion criteria included empirical research articles (quantitative, qualitative, or mixed methods), case studies, evaluations, experience reports, observational studies, systematic reviews, scoping reviews, meta-analyses, and relevant conference papers that reported on the application and impact of AI technologies in clinical documentation. We focused on studies involving NLP, ML, SR, and other AI technologies used in various clinical settings, including inpatient units, emergency departments, and outpatient clinics. We considered studies published within the last five years to ensure we captured recent advancements in the field and included only those published in English or with an English translation available.

Exclusion criteria filtered out studies not centered on clinical documentation, applications of AI unrelated to documentation (e.g., diagnostic tools, medical imaging), studies conducted in non-clinical settings, and studies with outcomes not related to documentation practices. We also excluded studies published before 2019 or those published in languages other than English without available translations. 

Pilot Testing

Prior to finalizing our methods, we conducted pilot testing with a small subset of articles to fine-tune our data extraction process. This helped us find any potential challenges in our review process and refine our research question and inclusion/exclusion criteria.

Data Extraction and Review Process

Multiple reviewers took part in data extraction. Each article was independently read to determine its relevance to our research question. A qualitative assessment was carried out which allowed us to capture subtle insights from the literature. Table 2 shows the data which was extracted from each article.

**Table 2:** Data extraction and review summary of AI in clinical documentation AI: artificial intelligence

Data extracted	Details
Study characteristics	Authors, publication year, study design
Clinical setting	Inpatient, outpatient, emergency department
Type of AI technology	Natural language processing, speech recognition, machine learning
Key findings	Impact on documentation accuracy and efficiency
Benefits and challenges	Reported benefits and challenges
Recommendations	Future research and practice recommendations

We narrowed down the articles to 36 in total after applying our inclusion and exclusion criteria. The reviewers conducted an inter-rater reliability assessment, achieving a Cohen's kappa score of 1.0, indicating perfect agreement in our initial screening and selection process. We resolved minor discrepancies through consensus, to ensure the integrity of the data extraction process and a thorough understanding of the literature.

Preferred Reporting Items for Systematic Reviews and Meta-Analyses (PRISMA) Guidelines

**Figure 1:**
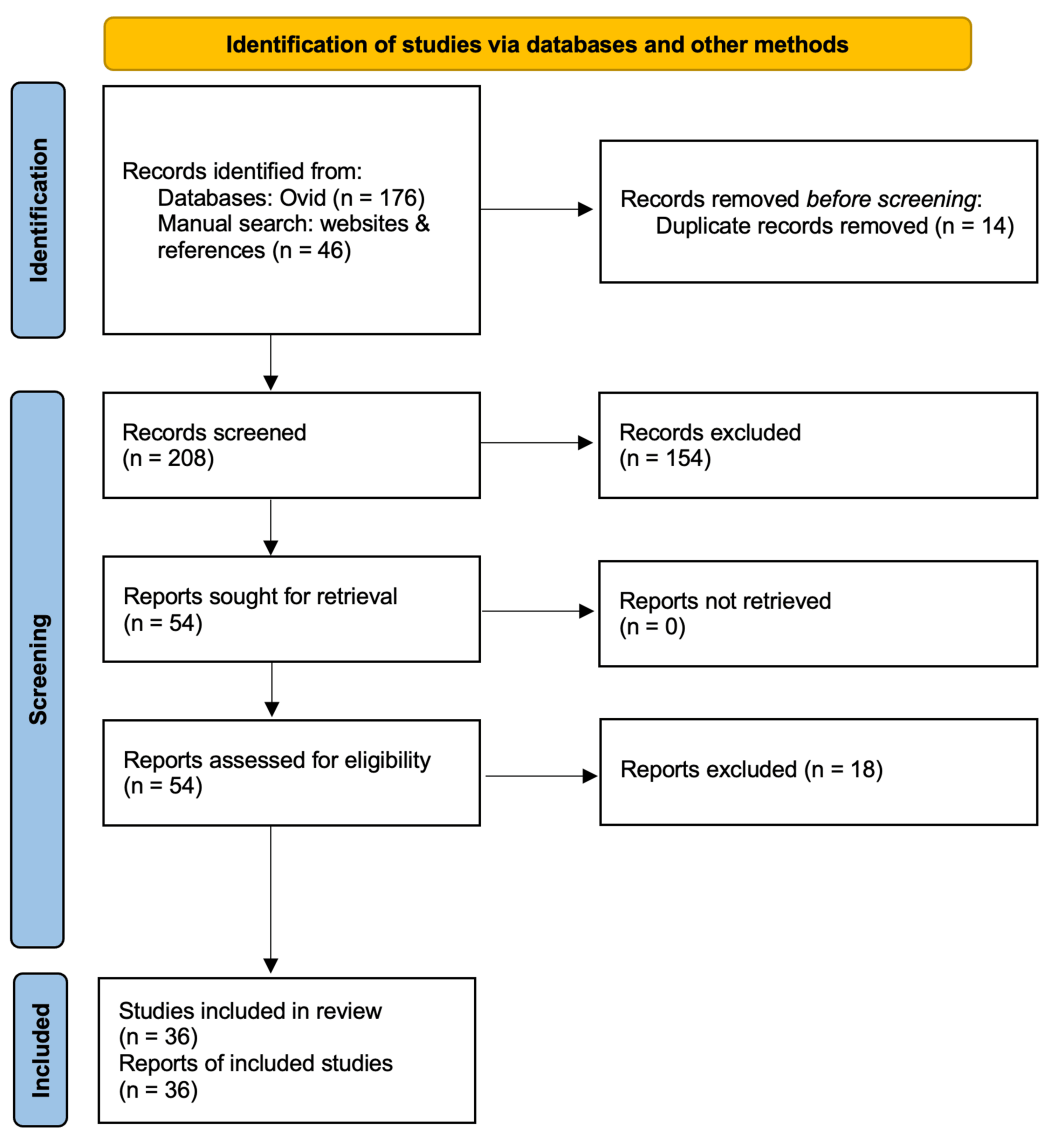
PRISMA flow diagram for the scoping review on evaluating the impact of AI on clinical documentation efficiency and accuracy across clinical settings PRISMA: Preferred Reporting Items for Systematic Reviews and Meta-Analyses; AI: artificial intelligence Reference: [31]

We followed the PRISMA checklist to guide our review process, ensuring a comprehensive and systematic examination of the current landscape of AI technologies in clinical documentation across various clinical settings. A PRISMA flowchart illustrating the selection process is included (Figure 1).

## Review

Overview of studies on AI in clinical documentation

The studies reviewed span various healthcare settings and explore different aspects of using AI, specifically LLMs, NLP, ML, and SR, in clinical documentation. This broad analysis includes observational studies, scoping reviews, systematic studies, and experience reports. Table 3 shows findings from these articles which are relevant to this review. Together, these studies provide a thorough overview of the current state, benefits, challenges, and future directions of AI-driven clinical documentation.

**Table 3:** Summary of key findings in AI-enhanced clinical documentation LLMs: large language models; AI: artificial intelligence

Aspect	Finding	Citation
Efficiency and user experience	Speech recognition improves workflow efficiency but requires editing by clinicians for accuracy.	[7,20]
	LLMs like GPT-4 make discharge summaries more readable, even though they still require clinicians to edit them afterwards.	[3,5,17,19,23,25,29]
	LLMs facilitate better communication between physician and patient. It also improves medical training.	[3,5,17,25]
	Ambient AI scribes reduce clerical workload, by reducing the time clinicians spend documenting ward rounds. This improves clinician efficiency and patient interaction.	[32]
	Speech recognition reduces documentation time and improves workflow efficiency in various clinical settings.	[7,20]
Accuracy and error management	LLM-generated summaries sometimes contain misinterpretations, certainty illusions, fabricated information, and attribute errors. This is why clinicians may still be needed to review them.	[11,15,23]
	Speech recognition systems have been shown to improve documentation time but still need significant editing to ensure the reliability of its content.	[7,20,24]
	Various studies highlight that LLMs require careful review to ensure clinical accuracy and prevent misinformation.	[11,23,24,30]
Clinical utility and safety	Integrating LLMs into workflows can reduce clinician documentation burden, though this may come with its own risks such as fabricated information.	[11,25,29,32,33]
	LLMs can generate patient-friendly discharge summaries, enhancing patient-centered care.	[3,5]
	LLMs improve medical documentation but still require human oversight to ensure patient safety.	[4,15,32,34,35]
	AI-driven documentation tools like ambient scribes enhance clinical utility by reducing time spent on clerical tasks.	[32,36]
Patient-centered care	AI enhances patient health literacy and treatment adherence by providing information in an easier-to-understand language.	[3,5,8,32,35]
	Patient-friendly discharge summaries created using LLMs show improvements in readability and understandability.	[3,5,35]
	Ambient AI scribes allow clinicians to focus more on patient care by reducing the amount of clerical work. Patient-physician interactions are then improved.	[32,36]
Liability and ethical considerations	Legal uncertainties exist regarding liability for AI-generated medical errors, necessitating clear regulations.	[33,34]
	Ethical considerations such as data privacy and model interpretability are crucial for safe AI implementation.	[17,33,34]
	Balancing technical benefits with ethical considerations is essential, requiring more empirical research on LLMs’ clinical utility.	[17,34]

Key findings

Efficiency of AI Documentation

SR has been shown to be beneficial in reducing documentation time and improving workflow efficiency in various clinical settings. Many clinicians find this technology satisfactory and believe it improves efficiency [7,20].

Accuracy and Error Management

GPT-4 generated more accurate summaries compared to GPT-3.5 Turbo, with fewer inaccuracies and hallucinations, but errors like omissions were still a concern. Studies also found that LLM-generated summaries sometimes contain misinterpretations, certainty illusions, fabricated information, and attribute errors [6,11,15,23,29]. While SR have been shown to improve documentation speed, they still require significant editing to correct errors [7,20].

Clinical Utility and Safety

Integrating LLMs into clinical workflows can help reduce the documentation burden on clinicians and allow more time for patient care, though fabricated information poses safety risks [4,11,25,29,32,33].

Patient-Centered Care

Patient-friendly discharge summaries created using LLMs have shown improvements in readability and understandability [3,5,8,34,35]. This can then improve patient health literacy and even treatment adherence. Ambient AI scribes have improved patient-physician interactions by reducing clerical work and allowing physicians to focus more on patient care [32,36].

Legal and Ethical Considerations

There will always be concerns surrounding AI as it is implemented into healthcare systems; therefore, regulations are needed to address ongoing legal uncertainties about liability for medical errors caused by AI systems. Ethical considerations such as data privacy are also crucial for the safe implementation of AI in healthcare [17,33,34].

Comparative studies and innovations

Studies comparing AI-generated summaries with those by senior internal medicine residents found similar performance levels, suggesting AI's potential utility in medical documentation [37]. LLM-generated notes from doctor-patient conversations are often preferred nearly as much as human-written notes, indicating their potential for automated clinical documentation [15].

Modular summarization techniques like CLUSTER2SENT and fine-tuning transformer models (e.g., BART) show promise in generating high-quality clinical summaries from conversation transcripts [24,30]. Pre-trained models like BERTSUM and BART have been effectively used for summarizing medical dialogues, with GPT-3.5 generating more comprehensible summaries according to human evaluators [29].

Implementation challenges

Technical improvements and customization are required for the effective integration of AI tools with existing EHR systems, as this remains a challenge [19,36].

To ensure the maximal benefit of these AI technologies are obtained, extensive training of healthcare professionals is required. Only then will there be adequate adoption of AI tools in clinical practice. Studies highlight the importance of providing adequate training to clinicians to maximize the benefits of AI technologies [9,21]. Early adopters have reported improvements in documentation efficiency and accuracy after proper training [27].

Discussion

**Figure 2:**
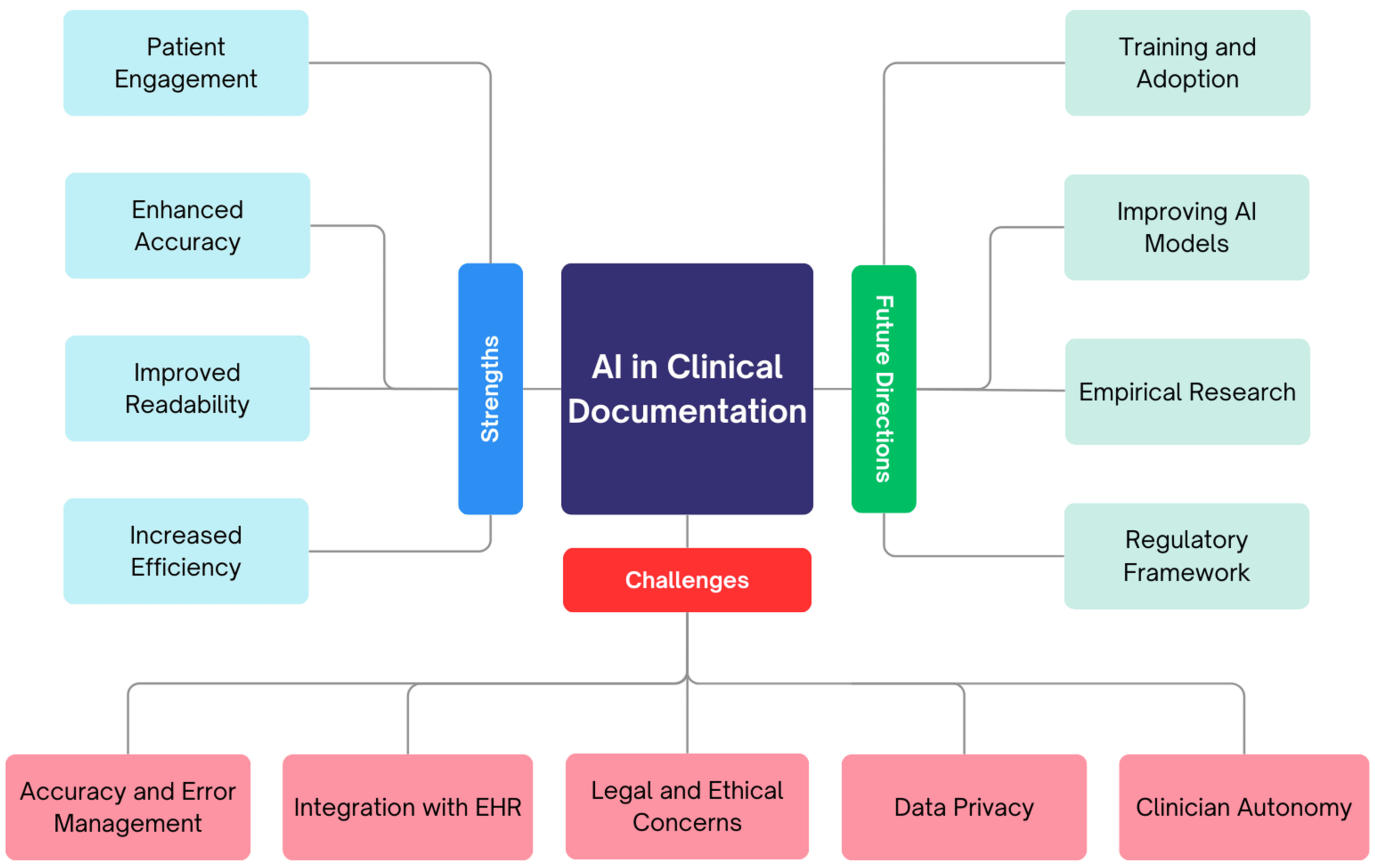
Outline of the strengths, challenges, and future directions of AI in clinical documentation AI: artificial intelligence

Several studies show that AI in clinical documentation can reduce the administrative burden on healthcare professionals. Today, this technology is becoming increasingly integrated into clinical settings and is already positively affecting staff and patients in some of the areas it is being used. It does this while keeping the accuracy and integrity of records. The result is reduced clinician workload and better workflow efficiency through increased speed of documentation and less administrative work [7,8,18,20,36]. We will also discuss some of the challenges and future potential and hope for AI in clinical documentation. Figure 2 provides an outline of the strengths, challenges, and future directions of AI in clinical documentation.

Strengths of AI in Clinical Documentation

One of the first strengths we will explore is increased efficiency in documentation. AI can complete tasks and process data faster than humans, and we see this positively impacting workflow efficiency, through a reduction in documentation time [19,20,27]. Reducing the time clinicians spend on documentation allows us to focus more on patient care [7,18,20,36].

One benefit for the patients is having a better understanding of the documents given to them by health institutions. In studies focused on AI-generated clinical summaries, it is clear that AI can enhance the readability and understandability of these documents, improving patient engagement and adherence to treatment [3,5,8,35]. Good readability is crucial for patient care, where clear communication can lead to better health outcomes and patient satisfaction [3,5]. LLMs have shown the potential to simplify complex medical jargon, making it easier for patients to understand their health status and follow medical advice [5].

Moreover, AI can produce comprehensive and accurate clinical summaries when effectively managed. Studies comparing AI-generated content with human experts have shown that AI can match or even exceed human accuracy [6,11,15,19,37]. There can also be errors in manual documentation, and in comparison studies between GPT-4 and GPT-3.5, we see the potential of AI to reduce these types of errors; however, AI does produce errors of its own [6,11,23]. Even though AI systems have been found to improve the speed and accuracy of clinical documentation, they will still require human oversight to ensure those errors do not occur [19,24,27].

Challenges With AI in Clinical Documentation 

One significant challenge is error management. Errors such as hallucinations (factually incorrect), omissions (leaving out important information), and fabricated information in AI-generated summaries pose significant risks and require rigorous validation and continuous oversight by clinicians [6,11,23]. For instance, studies have noted that while GPT-4 shows improvement in documentation, it still introduces errors that can affect clinical documentation quality and, consequently, clinical outcomes [6,14,16,26]. The main concern with these errors is their potential to compromise patient safety and treatment efficacy [33]. We therefore need to ensure that we have extensive error management strategies when integrating AI systems.

Effective integration of AI tools with existing EHR systems remains another challenge, requiring ongoing technical improvements and customization [19,36]. This is important as studies have highlighted the need for seamless integration to maximize the utility of AI in clinical documentation [36].

Bias and inequality are additional concerns. It is therefore important to address these biases to ensure fairness and equity in clinical documentation [17]. AI systems can perpetuate biases present in the data they are trained on, leading to unequal documentation outcomes. To prevent disadvantages to certain patient groups, there must be strategies in place to mitigate these biases [11,17]. 

Furthermore, determining accountability for decisions made by AI systems in clinical documentation is not clear-cut. There is a need for clear guidelines on the responsibilities of developers, healthcare providers, and institutions in the context of AI usage for clinical documentation [34]. Studies have called for frameworks that delineate the accountability of different stakeholders involved in the deployment and use of AI in clinical documentation [32,34]. 

There must always be measures in place to prevent unauthorized access and breaches. Without these measures, we may lose patient trust and undermine the integrity of clinical documentation systems. Hence, it is important that we comply with legal standards and ensure that the AI systems in use prioritize data security [15,17].

There are also healthcare providers who are hesitant to accept and trust AI systems. Efforts to improve the explainability and transparency of AI decision-making processes are crucial for their widespread adoption in healthcare [17,35]. Clinicians will begin to trust this technology as long as it maintains transparency. Only then will AI be able to facilitate more effective clinical documentation processes.

Balancing the use of AI with clinician autonomy is essential. AI should augment, rather than replace, clinical judgment to ensure that healthcare providers remain in control of clinical documentation decisions [34]. Maintaining this balance can help integrate AI into clinical documentation practice without undermining the expertise and decision-making capabilities of healthcare professionals [36].

Future Directions and Insights

There are several key areas in which research and development can focus on to improve AI in clinical documentation. Future models should be more reliable. ML can make this possible by allowing AI models to better understand context in medical language over time [2,5].

For a deeper understanding of AI in clinical documentation, further studies will need to be done. More randomized controlled trials, observational studies, and longitudinal studies are needed to assess clinical outcomes. Rigorous empirical research will provide robust evidence to support the integration of AI in clinical practice [17,32].

It is vital to set up clear legal and ethical guidelines to manage the use of AI in healthcare, particularly concerning liability for errors and data privacy concerns [15,34]. These frameworks should also include guidelines for the ethical use of patient data in training AI models and ensuring transparency in AI decision-making processes [17].

Over time, the needs of healthcare providers and patients are always evolving. Therefore, for these AI systems to be adaptable, user feedback would be crucial where clinicians can report errors and improvements can be made in real-time [8,11].

While AI has some reported benefits in documentation, for it to be used correctly, healthcare staff will need adequate training. If this is not done, the implementation of AI tools will not be successful. Education and support for staff through comprehensive training programs will then be of utmost importance to effectively use the technology [9,14].

Building patient trust in AI is crucial. Ensuring transparency in how AI tools are used and involving patients in the review process can enhance trust and acceptance. Efforts to involve patients in the development and refinement of AI tools can also help ensure that these technologies meet their needs and expectations [36].

For health institutions to implement AI systems successfully, the cost-effectiveness of their use will need to be assessed. Reduced documentation time and increased efficiency can save money [32]. However, a thorough analysis of the long-term financial benefits of AI will need to be done.

Limitations

Several limitations were encountered while performing this scoping review. For instance, we acknowledge that most of the articles included are experience reports, cross-sectional studies, or observational studies. There is a lack of longitudinal studies, which limit understanding of AI's long-term impacts. With adequate funding and the integration of AI systems into healthcare, future research can focus on cohort studies that can determine the long-term effects of using AI in healthcare and assess patient outcomes. Also, due to the nature of our scoping review, we have not broadly covered all AI technology currently in use, as well as emerging AI tools such as predictive analytics and computer vision. We focused on NLP, SR, and ML and their use in various clinical settings, mainly in developed countries, and we also focused on studies published in English. This introduces a cultural and geographical bias; therefore, it is crucial to conduct research in diverse contexts to understand the impact of AI in healthcare globally. Lastly, to review the latest trends and advancements, we only included articles from the past five years. Research into older studies can provide some historical context and insights into the evolution of AI in clinical documentation.

## Conclusions

AI has significant potential to reshape clinical documentation through the enhancement of efficiency, accuracy, and patient engagement. This can be made possible by utilizing LLMs and various other AI technologies. We will see a reduction in documentation time, improved readability, and better patient-centered care. However, several key challenges must be addressed first. Accuracy and efficiency are the main concerns. We've observed that AI-generated summaries and progress notes sometimes contain errors, known as hallucinations and omissions. Therefore, for this process to be validated, it still requires oversight by clinicians. A comprehensive regulatory framework is therefore needed to address these errors, as there are legal and ethical concerns. Additionally, effective integration of EHRs must make full use of the various AI tools available.

Later efforts should aim at improving AI models by addressing biases, creating better prompt engineering, and developing robust evaluation metrics. More research, such as randomized controlled trials and observational studies, are necessary to improve the validity of AI in healthcare. Clear legal and ethical guidelines will help manage the risks associated with AI use, ensuring data privacy and model interpretability. Continuous evaluation and adaptation of AI tools are essential to meet the evolving needs of healthcare providers and patients. Collaboration between clinicians, AI developers, and policymakers is important to take full advantage of in AI in healthcare. By focusing on these areas, the healthcare industry can effectively leverage AI to reduce the documentation burden on clinicians, enhance patient care, and improve overall healthcare delivery. The path forward requires a balanced approach that integrates technical innovation with ethical and regulatory considerations, ensuring that AI technologies are implemented safely and effectively to support the goals of modern healthcare.
